# The association between serum vitamin D and body composition in South African HIV-infected women

**DOI:** 10.4102/sajhivmed.v22i1.1284

**Published:** 2021-09-30

**Authors:** Samuel Mwango, Janet Carboo, Christa Ellis, Marike Cockeran, Carina M.C. Mels, Herculina S. Kruger

**Affiliations:** 1 Centre of Excellence for Nutrition, Faculty of Health Sciences, North-West University, Potchefstroom, South Africa; 2 Statistics Consultation Service, Faculty of Natural Sciences, North-West University, Potchefstroom, South Africa; 3 Department of Physiology, Faculty of Health Sciences, North-West University, Potchefstroom, South Africa; 4 Medical Research Council, Hypertension and Cardiovascular Disease Research Unit, Faculty of Health Sciences, North-West University, Potchefstroom, South Africa

**Keywords:** Vitamin D, postmenopausal, adiposity, bone mineral density, Africa, HIV/AIDS, ART

## Abstract

**Background:**

HIV and antiretroviral therapy (ART) alter vitamin D metabolism, and may be associated with bone loss.

**Objectives:**

The aim of this study was to determine the association between serum 25-hydroxyvitamin D (25(OH)D) and body composition in postmenopausal South African women living with HIV and on ART.

**Method:**

In this 2-year longitudinal study on 120 women conducted in the North West province of South Africa, serum 25(OH)D concentration, bone mineral density (BMD) at three sites, lean mass and percentage of body fat (%BF) were measured by dual-energy X-ray absorptiometry (DXA). Multivariable linear mixed models were used to assess the association between serum 25(OH)D and body composition over 2 years. Linear mixed models were also used to determine the longitudinal association between lean mass, %BF and BMD.

**Results:**

Vitamin D deficiency and insufficiency increased from baseline (10.2% and 19.5%) to 11.5% and 37.5%, respectively, after 2 years. Serum 25(OH)D decreased significantly, however, with a small effect size of 0.39 (*P* = 0.001), whilst total BMD (effect size 0.03, *P* = 0.02) and left hip femoral neck (FN) BMD (effect size 0.06, *P* = 0.0001) had significant small increases, whereas total spine BMD did not change over the 2 years. Serum 25(OH)D had no association with any BMD outcomes. Lean mass had a stronger positive association with total spine and left FN BMD than %BF.

**Conclusion:**

Serum 25(OH)D was not associated with any BMD outcomes. Maintenance of lean mass could be important in preventing bone loss in this vulnerable group; however, longer follow-up may be necessary to confirm the association.

## Background

Vitamin D is a pro-hormone primarily known for its role in bone development through calcium and phosphorus homeostasis.^1^ It is acquired from dietary sources that include fish, egg yolk, liver and vitamin D fortified foods; however, cutaneous synthesis of vitamin D contributes to more than 90% of the requirements.^2^ Epidemiological studies across the world indicate a close association between vitamin D deficiency and chronic diseases, such as bone metabolic disorders, tumours, cardiovascular diseases and diabetes mellitus.^3^ The prevalence rate of vitamin D deficiency worldwide varies between 30% and 93%, and is a growing public health concern amongst all age categories and ethnic groups.^4,5,6^ Hypovitaminosis D is also a prevalent disorder in low- and middle-income countries.^7^ Durazo-Arvizu et al.^8^ reported a higher prevalence rate of vitamin D deficiency amongst South African men and women aged 25–45 years compared with groups from Ghana, Jamaica and Seychelles. Many factors, including skin pigmentation, sun exposure, sex and low dietary intake, are known to be associated with the vitamin D status. In South Africa, the country with the largest proportion of people living with HIV, along with the largest antiretroviral therapy (ART) programme, the prevalence of vitamin D deficiency may be even worse because of the known relationship between HIV infection and vitamin D status.^9,10,11^

In South Africa, a total of 7.5 million people were reported to be living with HIV in 2019. This makes South Africa a country with the highest burden of HIV infection in the world, with a prevalence rate of 19.0%, in the age group 15 to 49 years.^12^ Furthermore, South Africa has the largest HIV treatment programme, with 3.9 million people initiated on ART in 2016.^13^ HIV management strategies coupled with the use of ART have shifted the trends of HIV from an inevitably fatal disease to a chronic infection.^14^ Chronic HIV infection and exposure to ART are associated with altered vitamin D metabolism, decreased bone mineral density (BMD) and increased fracture risk.^15,16,17^ A longitudinal study in postmenopausal women showed that women living with HIV had greater rates of bone loss at the spine and forearm than HIV-negative postmenopausal women.^18^ Body composition alteration (lipodystrophy) is a common phenomenon in people living with HIV.^19^ Furthermore, body composition is an important factor that influences vitamin D status directly or indirectly through interactions with other factors, such as age, sex, skin pigmentation, menopausal status, chronic diseases and medications.^20^ An inverse correlation has been established between vitamin D status and adiposity in adults.^21,22^ Low- and middle-income countries including South Africa, are experiencing nutrition-related non-communicable diseases resulting from nutrition transition and consequent body composition changes towards increased adiposity.^23^ The South African National Health and Nutrition Examination Survey-1 (SANHANES-1) of 2012 and the South African Demographic and Health Survey of 2016 confirmed the high rates of overweight and obesity in adult women to be 27% and 41%, respectively.^24,25^

This research study aimed at contributing knowledge on the association between serum 25-hydroxyvitamin D (25(OH)D) and body composition amongst HIV-infected postmenopausal women. To our knowledge, this is the first study to involve the target population in South Africa, which has an increased risk of both vitamin D deficiency and poor bone health.

The findings from the study can potentially inform treatment and care of HIV-infected postmenopausal women.

## Materials and methods

### Study design, setting and participants

This was a longitudinal study that used data measured in 2017 at baseline, 1-year (2018) and 2-year follow-up (2019) from a larger prospective bone study at the Centre of Excellence for Nutrition (CEN) at the North-West University in Potchefstroom, South Africa. Study participants were recruited from public outpatient ART clinics. Participants who met the inclusion criteria were invited individually to participate and receive information about the project. Those who accepted were included through consecutive convenience sampling. All measurements were performed at the metabolic unit of the North-West University. Signed informed consent was obtained from all participants.

**FIGURE 1:**
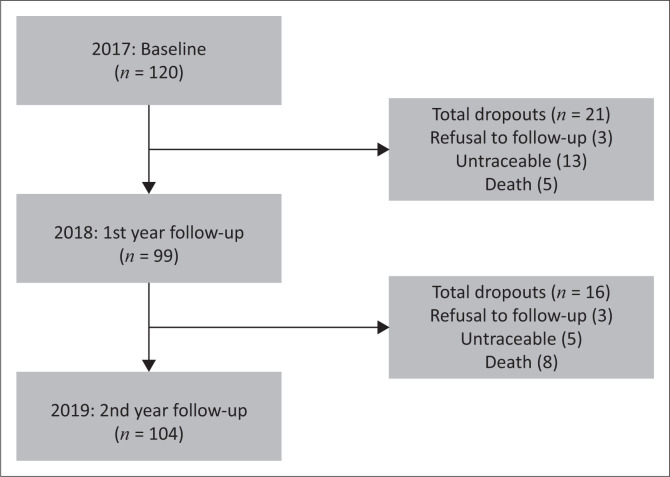
Participant flowchart and reasons for loss to follow-up.

The study involved South African postmenopausal women living with HIV and *already* on ART. Most participants were on ART regimen-1 for the duration of the study (tenofovir [TDF], emtricitabine [FTC] and efavirenz [EFV]) as a combined antiretroviral therapy (cART). This sub-study was performed on the available participants from the larger study, which included 120 postmenopausal women. The flowchart of participants shows the number of study participants at each measurement date and reasons for loss to follow-up, as shown in Figure 1.

A power calculation for the primary analysis of this study (multivariable regression analysis) indicated that a sample size of at least 262 would be necessary at a ß of 0.4 for the primary exposure, a power of 0.95 and an α of 0.05.^26^ It is therefore possible that the sample size may be too small to show an association even if there was one; however, it was not possible to recruit more women, because most HIV-infected women from these clinics were premenopausal. We therefore analysed and interpreted the available data with caution.

The inclusion criteria included HIV-infected postmenopausal women on ART. ‘Postmenopausal’ was defined as the self-reported absence of menses for at least 6 months prior to the study. The exclusion criteria included use of anti-osteoporotic agents (corticosteroids, thyroid medication initiated during the previous year, anti-vitamin K agents, diuretics, anti-epileptic drugs and β-blockers), calcium and vitamin D supplements, or antacids containing calcium according to their medical records or self-report. Chronic liver disease, chronic obstructive pulmonary disease, chronic renal disease, immobility, rheumatoid arthritis, gastrectomy, malabsorption syndromes, diagnosed diabetes mellitus, history of metabolic bone disease, high alcohol consumption (≥ 3 units/day) and history of a fracture within the last 6 months were also part of the exclusion criteria. Women with severely low BMD at the hip (T-score < –3) detected at baseline were not enrolled but referred for medical treatment. According to the World Health Organization (WHO), a T-score is the distance in the number of standard deviations from the mean BMD for a young reference group based on data from a white reference population. A T-score of –2.5 and below indicates osteoporosis, and –1.0 to –2.5 indicates osteopenia, whereas a T-score of –1.0 and above represents normal BMD.^27^

### Measurements

#### Socio-demographic and health information

Information on participants’ socio-demographic and health status was collected using an interviewer-administered structured questionnaire. The following information was captured: age, education level, housing, occupation, smoking, alcohol consumption, chronic medication use (including ART), year of first HIV diagnosis and year of ART initiation. All women brought their medication along to the study site to be recorded.

#### Anthropometric measurements

Anthropometric measurements included height and weight, and were performed by trained postgraduate Nutrition and Dietetics students. The measurements were conducted on participants without shoes and with minimal clothing. Weight and height were measured using a calibrated digital scale and attached stadiometer (Seca 264, Hamburg, Germany), following standard procedures.^28^ Body mass index (BMI) was calculated by dividing weight in kilograms by height in metres squared. BMI cut-off points were < 18.5 for underweight, 18.5 to < 25 for normal weight, 25.0 to < 30 for overweight and ≥ 30 kg/m^2^ for obesity.

**Physical activity:** Physical activity was assessed by a trained fieldworker using the Global Physical Activity questionnaire (GPAQ) recommended by the WHO.^29^ The questionnaire gathered information on physical activity performed during the previous 7 days in the following occupation: physical activity, transport-related physical activity and physical activity during leisure time. The time spent during the various physical activity domains in terms of frequency (days per week) and duration (minutes per day) was estimated.

**Bone mineral density, lean mass and fat mass:** Bone mineral density, lean mass and fat mass were measured at all three time points by a registered radiographer using dual-energy X-ray absorptiometry (DXA) with the default Hologic settings (Hologic Discovery W, APEX system software version 2.3.1). BMD at total body, lumbar spine and left femoral neck (FN) of the hip was measured in g/cm². Participants were asked to remove all jewellery and were provided with cotton gowns without any metal trimmings to wear during the DXA measurement.

**Serum vitamin D and parathyroid hormone concentration:** Fasting blood samples of 5 mL were collected in serum tubes by a registered nurse, were centrifuged immediately and stored in a bio-freezer at –80 °C until samples of all the study participants were collected. Serum 25(OH)D and parathyroid hormone (PTH) were measured by electrochemiluminescence (Cobas e411, Roche, Basel, Switzerland). Samples collected for each time point were analysed together in one batch with the same controls.

**Dietary data:** Dietary intakes were assessed at baseline and at 1-year follow-up visits in 2018 by trained fieldworkers using a validated quantitative food frequency questionnaire, food models and a validated food picture book to estimate portion sizes.^30,31^ Portion sizes, reported in household measures, were converted to weights. Energy and nutrient intakes (i.e. protein, fat, vitamin D and calcium) were calculated using food finder software based on the South African food composition database.^32^

### Statistical analysis

The distribution of the continuous variables was assessed by histograms, QQ plots and the Kolmogorov–Smirnov test. Descriptive statistics of socio-demographic data, physical activity, BMD at different sites, lean and fat mass, as well as serum 25(OH)D and PTH are presented as means and standard deviation (s.d.) for variables following a normal distribution or median and interquartile range (IQR) for non-normally distributed data. The percentage of participants with vitamin D deficiency and insufficiency were determined, where vitamin D deficiency was defined as serum 25(OH)D level < 20 ng/mL (50 nmol/L) and insufficiency as 20 ng/mL – 29 ng/mL (50 nmol/L – 74 nmol/L) according to the Endocrine Society and International Osteoporosis Foundation cut-offs.^33^

Pearson’s correlation coefficient (for data with a normal distribution) or Spearman’s rank order correlation coefficient (for data with a non-normal distribution) was calculated to examine the correlation between serum 25(OH)D and fat mass, lean mass and whole-body BMD, spine BMD and left FN of the hip BMD, as well as possible covariates (age, alcohol intake, calcium intake, physical activity level and number of years since the HIV diagnosis). Age correlated significantly with several variables and, therefore, Pearson partial correlation with adjustment for age was also performed. Variables with a significant correlation were entered in the linear mixed models (LMMs). Variables with a non-normal distribution were logarithmically transformed before they were entered in the LMM. Differences in exposure and outcomes between groups (smokers compared with non-smokers) were assessed using the Mann–Whitney test, because most variables had a non-normal distribution.

Linear mixed models were used with fat mass, lean mass and BMD, respectively, as the dependent variables (outcomes) and serum 25(OH)D as the primary exposure, with adjustment for variables identified to correlate with the exposure or outcomes (age, alcohol intake, calcium intake, and physical activity level). These models were also used with BMD as the dependent variable (outcome) and fat mass as well as lean mass as primary exposure, with adjustment for variables identified to correlate with the exposure or outcomes (age, alcohol intake, calcium intake and physical activity level). Separate models were used for whole-body BMD, spine BMD and left FN of the hip BMD. Time was treated as a fixed effect and participant as a random effect. The restricted maximum likelihood (REML) estimation method was used, and an unstructured covariance structure was specified.

An adapted version of Cohen’s d is used to indicate the practical significance of the differences between the means. The original formula for Cohen’s d for analysis of variance (ANOVA) is as follows:

$$d=\frac{\left|{\bar{x}}_{i}-{\bar{x}}_{j}\right|}{\sqrt{MSE}},$$ [Eqn 1]

where *d* denotes effect size, *ẋ*_i_ and *ẋ*_j_ are means of two samples and *MSE* is the mean standard error.^34^ In this case, however, the estimated marginal means, calculated as part of the LMM analyses, are used and the MSE is replaced by the sum of the covariance estimates. The interpretation guidelines indicate a small effect or practical non-significant d at an effect size of 0.2, an effect size of 0.5 indicates a medium effect or practical visible difference and an effect size of 0.8 indicates a large effect or practical significant difference. Statistical Package for Social Sciences (SPSS) software, version 26 (IBM Company, Armonk, New York, United States) was used for all analyses.

### Ethical considerations

The study was approved by the Health Research Ethics Committee of the North-West University (Project: NWU-00061-17-A1-02).

## Results

The study participants’ characteristics are summarised in Table 1. Their median duration of being diagnosed with HIV was 11 years [IQR 7–13 years], and the median duration of using ART was 10 years (IQR 5–13 years). Most participants (approximately 85%) remained on ART regimen 1 (TDF, FTC, and EFV) as a cART during the 2 years of observation. Combined vitamin D deficiency and insufficiency was estimated to be 29.7% at baseline, 42.9% at year 1 and 49.0% at year 2 of follow-up. Serum PTH concentrations showed an increased trend over time. Only a small proportion of study participants (10%) were current smokers, with a median of 2 (IQR 1–4) cigarettes per day. Only five (4.2%) participants were previous smokers. The median intake of calcium and vitamin D in the study participants was lower than the recommended dietary intake for women, 51–70 years old, of 1200 mg/d and 15 mcg/d, respectively.^35^

**TABLE 1:** Study participants’ characteristics from baseline to 2-year follow-up.

Variable	Baseline (2017) (*n* = 120)				Year 1 (2018) (*n* = 99)				Year 2 (2019) (*n* = 104)			
	Median	IQR	*n*	%	Median	IQR	*n*	%	Median	IQR	*n*	%
Age (years)	50	48–55	-	-	51	49–56	-	-	52	**50–57**	-	-
Weight (kg)	66.6	54.1–80.0	-	-	68.1	57.9–83.0	-	-	67.6	54.8–83.9	-	-
Height (cm)	156.6	151.5–161.0	-	-	157.5	152.3–161.5	-	-	156.7	151.8–160.4	-	-
BMI (kg/m^2^)	27.1	22.4–32.6	-	-	28.4	23.5–32.2	-	-	28.3	23.0–33.2	-	-
Underweight (BMI < 18.5 kg/m^2^)	-	-	10	8.3	-	-	4	4.0	**-**	**-**	7	6.73
Normal BMI (18.5 kg/m^2^ – 24.9 kg/m^2^)	-	-	37	30.8	-	-	31	31.3	**-**	**-**	29	27.88
Overweight (BMI 25 kg/m^2^ – 29.9 kg/m^2^)	-	-	27	22.5	-	-	23	23.2	**-**	**-**	26	25.00
Obese (BMI > 30 kg/m^2^) of the total	-	-	46	38.3	-	-	41	41.4	**-**	**-**	42	40.38
Waist circumference (cm)	85.0	76.0–97.3	-	-	88.6	76.9–96.6	-	-	90.8	76.7–101.0	-	-
Calf circumference (cm)	33.9	30.0–37.5	-	-	34.4	30.7–37.6	-	-	33.9 30.2–37.7		-	-
Total BMD (g/cm^2^)	1.04	0.95–1.12	-	-	1.04	0.98–1.14	-	-	1.03	0.96–1.11	-	-
Spine BMD (g/cm^2^)	0.84	0.75–0.95	-	-	0.88	0.79–0.98	-	-	0.87	0.78–0.97	-	-
Left hip FN BMD (g/cm^2^)	0.74	0.65–0.84	-	-	0.73	0.65–0.84	-	-	0.76	0.68–0.86	-	-
Lean mass (kg)	37.0	32.9–42.6	-	-	38.5	33.8–45.2	-	-	37.7	34.0–45.6	-	-
Percent body fat	38.6	33.8–43.6	-	-	39.1	33.3–42.8	-	-	39.9	33.4–43.6	-	-
Serum 25(OH)D (ng/mL) (deficiency < 20 ng/mL)	36.6	28.1–44.2	-	-	31.5	25.0–41.1	-	-	30.1	24.9–37.6	-	-
Parathyroid hormone (pg/mL) (normal range 10-55 pg/mL)	41.3	34.8–58.2	-	-	42.5	31.5–56.9	-	-	47.0	36.8–61.8	-	-
Calcium intake (mg/day) (recommended intake: 1200 mg/day)	668.3	413.2–911.9	-	-	517.6	352.3–794.1	-	-	-	-	-	-
Vitamin D intake (mcg/day) (recommended intake: 15 mcg/day)	4.99	3.09–6.78	-	-	4.44	2.53–7.80	-	-	-	-	-	-
Energy intake (kJ/day) (average energy allowance: 7980 kJ/day)	10247	7680–12 514	-	-	9330	6166–11 247	-	-	-	-	-	-
Fat intake (g/day) (recommended intake: 42-74 g/day)	76.6	50.0–100.0	-	-	57.1	44.5–84.2	-	-	-	-	-	-
Protein intake (g/day) (recommended intake: 46 g/day)	75.6	57.1–93.6			68.9	51.0–96.5	-	-	-	-	-	-
Alcohol intake (g/day)	0.0	0.0–0.86	-	-	0.0	0.0–1.3	-	-	-	-	-	-
PA (MET min/week)	2880	960–7190	-	-	2970	840–6740			3240	2040–4530	-	-
Sedentary time (min/day)	240	150–375	-	-	240	150–375			240	180–360	-	-
Vitamin D status†	-	-	-	-		-	-	-	-	-	-	-
Deficiency	-	-	12	10.2	-	-	14	14.3	-	-	12	11.50
Insufficiency	-	-	23	19.5	-	-	28	28.6	-	-	39	37.50
Sufficiency	-	-	83	70.3	-	-	56	57.1	-	-	53	51.00
Education	-			-	-			-	-			
No school/primary school	-	-	44	36.7	-	-	29	29.3	-	-	31	29.80
Grade 8-11	-	-	44	36.7	-	-	40	40.4	-	-	43	41.30
Grade 12	-	-	31	25.8	-	-	29	29.3	-	-	29	27.90
Tertiary	-	-	1	0.8	-	-	1	1.0	-	-	1	1.00
Income (per month)	-	-			-	-			-			
None	-		6	5	-		3	3	-		10	9.60
< R500.00 – R1000.00	-	-	31	25.8	-	-	21	21.2	-	-	15	14.40
R1001.00 – R6000.00	-	-	74	61.7	-	-	64	64.6	-	-	72	69.20
> R6000.00	-	-	9	7.5	-	-	11	11.1	-	-	7	6.70
Hypertension	-	-	55	45.8	-	-	46	46.5	-	-	48	46.20

BMI, body mass index; BMD, bone mineral density; FN, femoral neck; Ca, calcium; IQR, interquartile range; MET, metabolic equivalent; PA, physical activity; 25(OH)D, 25 hydroxyvitamin D.

† , Participant numbers vary because of missing values for some biochemical values.

Table 2 shows correlations between continuous variables according to the distribution of data. Serum 25(OH)D did not correlate with any of the BMD outcomes or total percent fat. Total percent body fat reported a moderate positive correlation with total spine BMD (*r* = 0.319; *P* < 0.0001) and left hip FN BMD (*r* = 0.408; *P* < 0.0001); however, it had a weak positive correlation with total BMD (*r* = 0.239; *P* = 0.009). Lean mass had a weak negative correlation with age (*r* = –0.219; *P* = 0.016) and a strong positive correlation with BMI (*r* = 0.824; *P* < 0.0001).

**TABLE 2:** Correlation between serum vitamin D, lifestyle, health and body composition variables: Pearson’s correlation between serum vitamin D and body composition variables.

Variable	Total spine BMD		Left hip BMD		Total BMD		Total percent age fat	
	*r*	*P*	*r*	*P*	*r*	*P*	*r*	*P*
Serum vitamin D (ng/mL)	−0.003	0.970	0.001	0.990	0.000	0.999	−0.072	0.437
Percent body fat (%)	0.319	< 0.0001	0.408	< 0.0001	0.239	0.009	-	-

BMD, bone mineral density.

A comparison between body composition outcomes and vitamin D status of smokers and non-smokers at baseline revealed no difference in total lean mass, total spine BMD, total BMD, total left hip FN BMD, BMI or vitamin D status; the only difference was in total percent body fat (*P* = 0.032). There was no correlation between serum 25(OH)D and PTH at baseline (*r* = –0.11, *P* = 0.24), after 1 year (*r* = –0.16, *P* = 0.12) or after 2 years of follow-up (*r* = –0.14, *P* = 0.17).

Variables with a non-normal distribution that showed significant correlations from the Spearman correlation were log transformed, and similar correlations were found. New significant correlations that were observed included a weak negative correlation between BMI and serum 25(OH)D (*r* = –0.189; *P* = 0.04). Furthermore, BMI had moderate positive correlations with total BMD, total spine BMD and left hip FN BMD (*P* < 0.0001).

Body mass index, however, had a strong correlation with total percent fat (*r* = 0.841; *P* < 0.0001). Age displayed a weak inverse correlation with total spine BMD (*r* = –0.242; *P* = 0.008) and a moderate inverse correlation with total BMD (*r* = –0.400; *P* < 0.0001) and left hip BMD (*r* = –0.346; *P* < 0.0001). Physical activity correlated positively although weakly with total BMD (*r* = 0.227; *P* = 0.014) and left hip FN BMD (*r* = 0.235; *P* = 0.011).

Lean mass had a moderate positive correlation with total spine BMD (*r* = 0.473; *P* < 0.0001), as well as total BMD (r = 0.429; *P* < 0.0001), and a strong positive correlation with left hip FN BMD (*r* = 0.563; *P* < 0.0001).

These results did not change after adjustment for age, except that the correlation between physical activity and total BMD was no longer significant (*r* = 0.177; *P* = 0.06).

### Changes of study exposure variable and body composition outcomes over a 2-year period

The changes were assessed over the three time points of measurements from baseline to 2-year follow-up.

**FIGURE 2:**
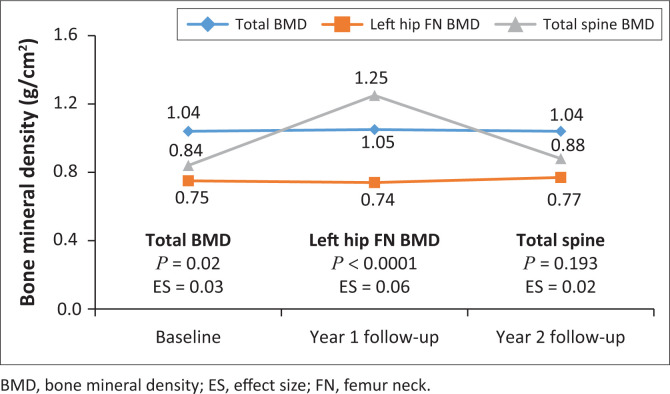
Bone mineral density changes over 2 years from baseline.

**FIGURE 3:**
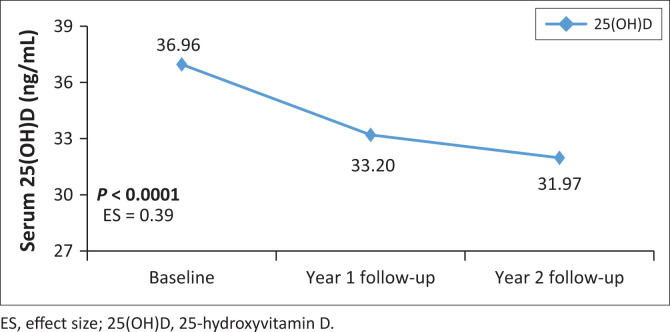
Serum 25-hydroxyvitamin D changes over 2 years from baseline.

**FIGURE 4:**
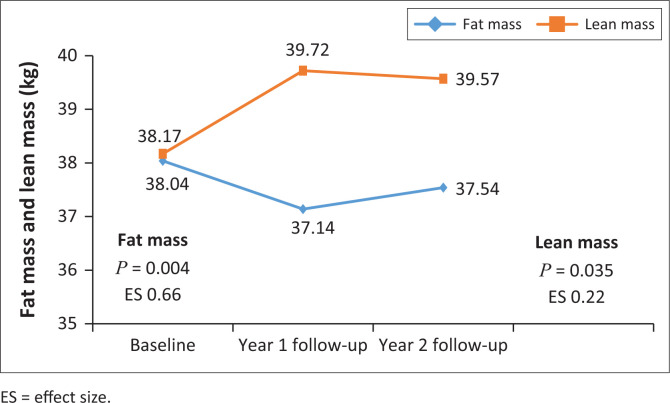
Lean and fat mass changes over 2 years from baseline.

Changes of main exposure and body composition outcomes are presented in Figures 2, 3 and 4. The serum 25(OH)D concentration declined from baseline through to year 2 follow-up (*P* < 0.0001) with an effect size of 0.39, indicating a practically visible difference. The Bonferroni pairwise comparison showed a significant decline in serum 25(OH)D concentration from baseline to year 1 follow-up and from baseline to year 2 follow-up. Total BMD increased from baseline to year 1 follow-up, but with practical non-significant difference (effect size = 0.03). Similarly, left hip FN BMD increased over 2 years; however, the difference was practically non-significant (effect size of 0.06). Changes in left hip FN BMD over the 2 years were between baseline and year 2 follow-up and year 1 and year 2 follow-up. There were no significant changes in PTH from baseline over the two years, although there was an increasing trend over time (Table 1). Lean mass had a practically non-significant difference between baseline and year 2 follow-up (effect size 0.02), whereas percent body fat mass had a practically visible difference over the two years (effect size 0.66).

### Association between serum vitamin D concentration and body composition over time

Linear mixed models were used to describe the association between serum 25(OH)D as the main exposure and body composition outcomes that included percent body fat, lean mass (logarithmically transformed), total BMD, lumbar spine BMD and left hip FN BMD. Associations between body composition components (fat mass, lean mass and BMD) were also determined by LMMs. Estimates of the associations were adjusted for education level, household income, age, physical activity, duration of ART and alcohol intake as potential confounders. Table 3 presents the associations. All models showed an improvement in goodness of fit after adding covariates to the crude models, as demonstrated by the Schwarz Bayesian information criterion (BIC).

**TABLE 3:** Correlation between serum vitamin D, lifestyle, health and body composition variables: Spearman’s correlations between lifestyle, demographic variables and body composition.

Variable	Lean mass		Total physical activity MET (min/week)		HIV duration (years)		Duration of ART use		Ca intake	
	*r*	*P*	*r*	*P*	*r*	*P*	*r*	*P*	*r*	*P*
Age (years)	−0.219*	0.016	−0.062	0.503	−0.042	0.648	−0.014	0.883	−0.016	0.864
BMI (kg/m^2^)	0.824**	< 0.0001	0.115	0.210	0.042	0.646	−0.017	0.852	−0.018	0.842
Physical activity (MET min/week)	0.147	0.108	-	-	−0.012	0.899	−0.051	0.578	0.004	0.966
HIV duration (years)	0.032	0.731	−0.012	0.899	-	-	0.856**	< 0.0001	−0.083	0.368
Duration of ART use (years)	−0.038	0.684	−0.051	0.578	0.856**	< 0.0001	-	-	−0.131	0.157

ART, antiretroviral therapy; BMI, body mass index; MET, metabolic equivalent.

* , significant correlation, *P* < 0.05;

** , significant correlation, *P* < 0.01.

The model with the lowest BIC was selected as the best model.

Serum 25(OH)D was neither associated with any of the three BMD outcomes, nor with percent body fat or lean mass. However, serum 25(OH)D showed a significant association with BMI in the unadjusted model (*P* = 0.024); however, there was no significant association after adjusting for education level, household income, age, physical activity and alcohol intake. There was a positive association between physical activity and left hip FN BMD. Age was consistently inversely associated with all body composition outcomes. Both percent fat mass and lean mass were associated with left hip FN BMD and total BMD, but not with total spine BMD (Table 4).

**TABLE 4:** Association between serum vitamin D and body composition and interdependent associations between body composition variables.

Variable	Total BMD		Total spine BMD		Left hip FN BMD		Percentage body fat		Lean mass	
	Mean estimate	*P*	Mean estimate	*P*	Mean estimate	*P*	Mean estimate	*P*	Mean estimate	*P*
**Association between serum vitamin D and body composition**										
Age (years)	−0.009	< 0.0001	0.068	0.031	−0.008	< 0.0001	−0.105	0.406	−0.004	0.032
Education level	0.021	0.836	0.137	0.467	−0.006	0.585	-	-	-	-
Household income	0.005	0.519	0.125	0.364	0.020	0.010	-	-	-	-
Duration of ART (years)	−0.011	0.545	0.214	0.511	−0.001	0.562	-	-	-	-
Alcohol intake (g)	0.001	0.415	−0.006	0.820	−0.0001	0.894	−0.004	0.943	0.0008	0.573
Physical activity (MET min/week)	0.032	0.067	−0.118	0.708	0.041	0.021	0.558	0.323	0.0001	0.994
Serum 25(OH)D (ng/mL)	−0.951	0.886	−0.003	0.784	−8.20	0.904	−0.035	0.404	−0.001	0.131
**Interdependent associations between %BF, lean mass and BMD**										
Age (years)	−0.009	< 0.0001	-	-	-	-	-	-	-	-
Education level	−0.001	0.898	0.141	0.440	−0.009	0.299	-		-	-
Household income	0.005	0.496	0.125	0.353	0.021	0.003	-		-	-
Duration of ART (years)	−0.0009	0.582	0.020	0.526	−0.001	0.482	-		-	-
Alcohol intake (g)	0.001	0.359	−0.007	0.772	−2.609	0.984	-		-	-
Physical activity (MET min/week)	0.027	0.112	−0.072	0.817	0.029	0.070	-		-	-
Percent body fat (%)	0.003	0.004	−0.031	0.147	0.006	< 0.0001	-		-	-
**Interdependent association between lean mass and BMD**										
Age (years)	−0.008	< 0.0001	0.067	0.032	−0.007	< 0.0001	-		-	-
Education level	0.001	0.911	0.128	0.487	−0.006	0.541	-		-	-
Household income	0.005	0.511	0.127	0.349	0.020	0.005	-		-	-
Duration of ART (years)	−0.001	0.530	0.020	0.528	−0.001	0.451	-		-	-
Alcohol intake (g/day)	0.001	0.399	−0.006	0.807	−0.0002	0.857	-		-	-
Physical activity (MET min/week)	0.033	0.051	−0.128	0.682	0.041	0.016	-		-	-
Lean mass (kg)	0.256	0.001	−0.258	0.854	0.301	< 0.0001	-		-	-

ART, antiretroviral therapy; BMD, bone mineral density; FN, femoral neck; MET, metabolic equivalent.

## Discussion

This 2-year longitudinal study focused on investigating the association between serum 25(OH)D and body composition (lean mass, fat mass and BMD) in South African postmenopausal women living with HIV and on ART. According to the Endocrine Society and International Osteoporosis Foundation cut-offs for vitamin D status by Valcour et al.,^33^ serum 25(OH)D insufficiency (deficiency and insufficiency) increased from 29.7% at baseline to 49.0% at year 2 of follow-up. Despite a significant decrease in serum 25(OH)D over time, there were practically no changes in BMD over the 2 years. There was no association between serum 25(OH)D concentration and total BMD, spine BMD, left hip FN BMD, lean mass or %BF consistently throughout the study period. From the LMMs on the interdependent associations between body composition components, lean mass and fat mass showed positive associations with total BMD and left hip FN BMD but not with total spine BMD. Lean mass proved to be stronger predictor of BMD than fat mass.

The study findings on vitamin D status (insufficiency and deficiency prevalence) correspond with those from some observational studies amongst postmenopausal women populations.^36,37,38^ Kuchuk et al.^39^ reported similar serum 25(OH)D deficiency levels in both summer and winter amongst postmenopausal women from 29 countries across the world. Serum 25(OH)D concentrations also declined during the 2-year study period in the Dallas Heart Study on a multi-ethnic adult population in Dallas, Texas, United States.^40^

Vitamin D is primarily known for an association with BMD via the homeostasis of calcium and phosphate.^41^

There was no association observed between serum 25(OH)D and total BMD, left hip FN BMD and total spine BMD over a follow-up period of two years. The study findings are in agreement with a number of studies, including randomised controlled trials (RCTs) with vitamin D supplementation.^42,43^ A cross-sectional study by Kamineni et al.^44^ in India amongst postmenopausal women revealed findings similar to this study, where no correlation was observed between serum 25(OH)D and BMD. However, a cross-sectional study amongst Chinese postmenopausal women found positive correlations between serum 25(OH)D and BMD outcomes at lumbar spine, total hip and FN.^45^ Differences between the findings of these studies and the current study could be partially explained by differences in menopause duration, duration of sunlight exposure amongst the women, genetic factors and ethnicity.^44^

Study observation period is likely another plausible reason for the lack of association between serum 25(OH) and BMD. Changes in BMD take a long time to manifest and, hence, the duration could have been inadequate to detect noticeable significant changes in this study. Hamill et al.^46^ observed a cohort of urban black HIV-infected South African premenopausal women over a period of 24 months. A significant finding was the attenuation of bone mass loss beyond 12 months up to 24 months in the HIV-infected group on ART. Although the participants were premenopausal women, this signifies that bone loss in HIV-infected patients within the first 12 months on ART could be transient. Our study’s participants showed a small, but significant, BMD increase at left FN BMD and total BMD, but no change at total spine BMD. Other authors had suggested a follow-up longer than 24 months to ascertain BMD changes.^46^ This study’s participants had an average ART use duration of 9 years, which might explain BMD stabilisation. Information about previous ART regimens could not be determined; however, it is likely that most women received first-line ART regimens at the public outpatient clinics and could have been switched to the present first-line regimen (TDF, FTC, EFV) soon after these fixed dose combinations became available in 2013.^47^ Therefore, most women have been exposed to TDF for three years or longer.

Despite the lack of association between serum 25(OH)D and BMD, we reported a decline in serum 25(OH)D levels from baseline to two years; however, the effect size was relatively small. This decrease may be related to the HIV status of the women. A high prevalence of vitamin D deficiency has been described amongst HIV-infected patients.^10^ The inflammatory processes associated with HIV infection and ART metabolism both alter vitamin D metabolism and may have been associated with the decline in serum 25(OH)D levels during the observation period.^48^

Very low serum 25(OH)D concentrations trigger PTH surge, which leads to bone demineralisation as a compensatory measure to maintain balance in serum calcium.^49^ The decline in serum 25(OH)D concentrations probably explains the small, although non-significant, progressive increase in PTH, which was probably insufficient to lead to a decline in BMD. In agreement with this study’s findings, Kota et al.^50^ reported no direct association between serum 25(OH)D and BMD. However, they found a stronger inverse association with serum 25(OH)D and PTH, indicating that low serum 25(OH)D level was indirectly associated with low BMD. Contrary findings to ours were reported in a 12-months longitudinal study amongst urban black South African premenopausal women. An increase in serum 25(OH)D regardless of the HIV status was reported over 12 months.^51^ The obvious difference between the two studies is age of the participants, where the study participants were postmenopausal women (mean age: 50 years), whereas the other study included premenopausal women.

Aging is a known risk factor for hypovitaminosis D through decreased vitamin D receptors and consequent alteration of vitamin D metabolism.^52^ We also found a non-significant inverse correlation between serum 25(OH)D concentration and age. A longitudinal study in Amsterdam reported a small decline in serum 25(OH)D levels in older participants (65–88 years) over 13 years, which is in agreement with the study findings.^53^ Poopedi et al.^54^ affirmed progressive fluctuations in serum 25(OH)D levels in a study amongst healthy adolescents over 10 years. These progressive changes in serum 25(OH)D levels observed in longitudinal studies, as well as this study, signify that single measurements are unreliable for determining associations between serum 25(OH)D and diseases.

This study reported no association between serum 25(OH)D and percent body fat or lean mass; however, there was a weak negative correlation with BMI. In agreement with this finding, there is a recent cross-sectional study amongst postmenopausal women, which reported an inverse correlation between serum 25(OH)D and anthropometric measurements, including BMI.^55^ BMI is used as a proxy for adiposity, and the sequestration theory provides a probable explanation for the inverse association displayed between body fat and serum 25(OH)D.^56^

In a cross-sectional study, however, Li et al.^45^ reported no association between BMI and serum 25(OH)D in Chinese postmenopausal women. The authors argued that their result was based on the unique body composition of the Chinese women, characterised by a lower prevalence of obesity than in other ethnic groups. This emphasises the role of ethnicity in the interaction between serum 25(OH)D and body composition. The study participants had a relatively high prevalence rate of overweight and obesity, which is in the range of 22.5% – 25.7% and 38.3% – 40.0%, respectively, over two years.

Previous observational studies have described other known determinants of BMD; however, the relative contribution of lean mass and fat mass to BMD remains a contentious subject. The data of this study showed that both lean mass and percent fat mass were positively associated with left hip FN BMD and total BMD, but not with total spine BMD. However, lean mass was a stronger predictor of BMD than fat mass. This result is a replication of findings from other observational studies amongst postmenopausal women.^57,58,59^ Similarly, a systematic review by Ho-Pham et al.^60^ proved lean mass to be a stronger determinant of BMD compared with fat mass. This signifies that the interdependent interactions between body composition components can be influenced by menopause status. The association between fat mass and BMD is probably mediated by the increased load placed on the skeleton, whereas lean mass influences BMD through increased mechanical stress mediated by muscle on the skeleton. Both mediators act as stimuli for osteogenesis.^61,62^

A significant proportion of the women in this study were overweight (22.5% and 25.7% at baseline and 2-year follow-up, respectively) and obese (38.3% at baseline and 40.0% at 2-year follow-up). We reported a quite unusual finding of a small, but significant, fat mass decline and lean mass increase in the women during the 2-year study period, in contrast with normal aging changes.^57^ The results of this study revealed a significant positive association between physical activity and left hip FN BMD, the site most prone to fracture in postmenopausal women. Such findings were reported previously in a Canadian retrospective study amongst postmenopausal women.^63^

Apart from serum 25(OH)D concentration, known risk factors for low BMD, such as dark skin, HIV, ART and advanced age (postmenopausal status), were present in the study participants. Physical activity levels were relatively good, signifying probable adequate exposure to sunlight and adequate stimuli to maintaining bone health. We further reported low exposure to lifestyle risk activities such as smoking and alcohol intake. These factors may further explain the small decrease in serum 25(OH)D levels and the preserved BMD over a follow-up period of two years. Diet is another known modifiable risk factor for optimal bone health, with the value of specific nutrients, such as dietary calcium and vitamin D, well established.^64^

The dietary calcium and vitamin D intake of participants were relatively low compared with dietary recommendations (1200 mg and 15 mcg, respectively, per day).^35^ These results are in line with those of previous studies amongst South African women.^65^ Foods are poor sources of vitamin D. Therefore, most of the daily input of vitamin D comes from cutaneous synthesis rather than from diet.^64^ In countries where foods are fortified with vitamin D, these foods are the major dietary source of vitamin D^64^; however, few foods are enriched with vitamin D in South Africa.^65^

## Recommendations

Urbanisation of black South African women may increase the risk of low bone mass because of low vitamin D status, low calcium intake and high bone turnover.^65^ Hence, there is a need for advising women on the intake of vitamin D and calcium. As the largest source of vitamin D is through cutaneous synthesis, an increase in outdoor activities and exposure to the sun may help to improve the vitamin D status.

## Limitations and strengths

Limitations and strengths existed in this study that need to be acknowledged. Firstly, this study used an observational design with limited control over other potential covariates, and no HIV-negative reference group was included. Secondly, all participants originated from the same locality in the North West province of South Africa and, therefore, generalisation of the findings to a wider population across South Africa and beyond should be performed cautiously. Thirdly, the sample size was relatively small, thereby undermining the power for subgroup analysis, that is, subgroups based on the vitamin D status. It was challenging to recruit more participants, because most HIV-infected women from the outpatient clinics were premenopausal. The findings of no statistically significant associations could, therefore, be because of type II error, and a larger sample could potentially have yielded a different result.

Regarding strengths, to the best of our knowledge, this is the first longitudinal study on an association between serum 25(OH)D and body composition in South African postmenopausal women living with HIV and on ART in South Africa. A longitudinal design provides stronger evidence of probable associations when compared with a cross-sectional study design. Future studies should consider all determinants of serum 25(OH)D concentration and body composition, such as duration of sunlight exposure, as well as dietary assessment over a longer duration.

## Conclusion

This 2-year longitudinal study indicates no association between serum 25(OH)D concentration and BMD over time. However, BMI was inversely associated with serum 25(OH)D concentration. In terms of the relative contribution of lean mass and fat mass to maintenance of BMD, lean mass is a stronger determinant of BMD than fat mass. South African postmenopausal women living with HIV and on ART maintained their BMD at all three sites over a period of two years. The study further suggests that BMD decreased with increasing age, whilst physical activity was protective against a decrease in BMD in this group of women. The results of this study may inform strategies to prevent fractures in postmenopausal women living with HIV. Increasing lean mass through physical activity could be important to preventing frailty and morbidity in this vulnerable group.
